# Are Small Effects the Indispensable Foundation for a Cumulative Psychological Science? A Reply to Götz et al. (2022)

**DOI:** 10.1177/17456916221100420

**Published:** 2022-09-20

**Authors:** Maximilian A. Primbs, Charlotte R. Pennington, Daniël Lakens, Miguel Alejandro A. Silan, Dwayne S. N. Lieck, Patrick S. Forscher, Erin M. Buchanan, Samuel J. Westwood

**Affiliations:** 1 Behavioural Science Institute, Radboud University; 2 School of Psychology, Aston University; 3 Institute of Health and Neurodevelopment, Aston University; 4 Industrial Engineering and Innovation Sciences, School of Innovation Sciences, Eindhoven University of Technology; 5 Annecy Behavioral Science Lab, Menthon-Saint-Bernard, France; 6 Development, Individual, Process, Handicap, and Education Research Unit, Université Lumière Lyon 2; 7 Social and Political Psychology Research Lab, University of the Philippines Diliman; 8 Independent Researcher; 9 Research and Innovation Division, Busara Center for Behavioral Economics, Nairobi, Kenya; 10 Analytics, Harrisburg University of Science and Technology; 11 Department of Psychology, School of Social Science, University of Westminster

**Keywords:** effect sizes, small effects, benchmarks, practical significance, statistical inference

## Abstract

In the January 2022 issue of *Perspectives*, Götz et al. argued that small effects are “the indispensable foundation for a cumulative psychological science.” They supported their argument by claiming that (a) psychology, like genetics, consists of complex phenomena explained by additive small effects; (b) psychological-research culture rewards large effects, which means small effects are being ignored; and (c) small effects become meaningful at scale and over time. We rebut these claims with three objections: First, the analogy between genetics and psychology is misleading; second, *p* values are the main currency for publication in psychology, meaning that any biases in the literature are (currently) caused by pressure to publish statistically significant results and not large effects; and third, claims regarding small effects as important and consequential must be supported by empirical evidence or, at least, a falsifiable line of reasoning. If accepted uncritically, we believe the arguments of Götz et al. could be used as a blanket justification for the importance of *any* and *all* “small” effects, thereby undermining best practices in effect-size interpretation. We end with guidance on evaluating effect sizes in relative, not absolute, terms.

In their recent commentary, Götz et al. (2022) argued that small effects are “the indispensable foundation for a cumulative psychological science.” Although we welcome their efforts to highlight the importance of reporting and interpreting effect sizes appropriately, we believe that some of their arguments have the potential to move us away from, and not toward, best practices in effect-size interpretation. Here we counter their arguments with three objections: First, the analogy between genetics and psychology is misleading; second, selection for statistical significance (*p* values) rather than selection for large effect sizes currently underpins publication bias; and third, statements that small effects may be important and consequential need to be supported by evidence and falsifiable reasoning rather than bald assertion. Furthermore, we disagree with Götz et al.’s assumption that “small” and “large” are meaningful categories outside of a particular theoretical and empirical context. We argue that effect sizes should be interpreted in relative and not absolute terms.

## Genetics Is Not a Useful Analogy for Psychology

Götz et al. (2022) began their line of reasoning by drawing an analogy between psychology and genetics: They argued that like the links between genes and behavior, psychological phenomena have multiple complex causal mechanisms, which means that the effects of any single mechanism are bound to be small. Although we broadly agree with the notion that psychological phenomena have multiple complex causal mechanisms, we are agnostic about the size of individual effects. Further, we argue that the analogy between genetics^1^ and psychology can confuse, more than advance, knowledge accumulation in psychological science for two main reasons.

First, in the study of genetics, there are a known and finite set of genes that can be measured accurately and quickly (see the Human Genome Project; Schmutz et al., 2004), but the same cannot be said for psychological phenomena. Even if it were possible to list all constructs of interest, it is not feasible to measure all of the constructs that psychologists might be interested in testing. In addition, psychologists are often interested in theoretically driven tests of predictions, which typically require specific experimental manipulations and often involve tests of interaction effects. Open data sets that are large enough to provide sufficient statistical power to detect small effects at a similar scale to genetics do not currently exist, and it is unlikely that psychologists will have the resources to create such data sets. This makes “psychological construct association studies” (to continue the analogy by Götz et al.) an impossible approach that will not enable researchers to efficiently generate knowledge about complex psychological phenomena.

Second, the scales used to measure psychological phenomena are much more coarse-grained than the measurement of genes, with many lacking the accuracy to reliably detect small effects (Flake et al., 2017; Fried, 2017). Until our measurement practices have improved, psychologists will not be able to distinguish measurement error from small effects. In reality, this makes it impossible to reliably study the small effects that Götz and colleagues hypothesized to be the indispensable foundation of a cumulative psychological science. Furthermore, even if measurement accuracy were perfect, we would still need to address the challenge of reliably distinguishing small effects of interest from “crud”—the notion that in large enough data sets in psychological science, all variables are correlated with each other (Meehl, 1990; Orben & Lakens, 2020; Vul et al., 2009). This is especially challenging because in some domains the crud factor is hypothesized to be as large as *r* = .10 (Ferguson, 2021).

In sum, we argue that caution is warranted when using genetics as an analogy for psychological science: Given the current state of affairs in psychological science, such as measurement imprecision, we simply do not know whether psychological phenomena are indeed caused by many additive small effects. One could even argue that given the limits of human information processing, small effects may not matter because they are simply not perceived.

## *P* Values (Not Effect Sizes) Currently Underpin Publication Bias and Questionable Research Practices

Contrary to what Götz et al. (2022) reported, Fanelli et al. (2017) did not find that “social scientific disciplines often cultivate publication cultures that favor or even demand large effects” (p. 206). They showed instead that small studies can overestimate effect sizes and that early studies in some fields have larger effects. Neither of these findings show any demand for large effects but rather the limitations of underpowered studies that lead to inflated, unreliable effect sizes. Instead, publication bias is underpinned by a preoccupation with *p* values: Effects that are statistically significant are published at a higher rate than nonsignificant effects in the traditional literature (see Fanelli, 2010; Scheel et al., 2021). Indeed, researchers often do not interpret effect sizes (Fritz et al., 2013; Motyl et al., 2017; Schäfer & Schwarz, 2019), and when requested by reviewers or editors to do so, it is usually on the basis of *justifying* whether certain “significant” effects matter rather than dismissing small effects altogether.

Moreover, Götz et al. (2022) argued that “the pressure to publish large effects is ‘dangerous’ because it . . . encourages practices that are likely to yield these inflated effects such as *p*-hacking, optional stopping, HARKing, and other questionable research practices” (p. 206). The smallest effect size that corresponds to a statistically significant result is a function of the alpha level and the sample size. Given a tradition of running small underpowered studies and selectively reporting statistically significant results (see Button et al., 2013; Szucs & Ioannidis, 2017), the mechanism Götz et al. described is, in fact, reversed: It is not a pressure to publish large effects that encourages questionable research practices (QRPs) but rather QRPs coupled with low statistical power that inflate effect sizes to reach “publishable” *p* values (see Stefan & Schönbrodt, 2022). Although we wholeheartedly agree with Götz et al. (2022) that effect sizes are important and should be evaluated in terms of their theoretical and practical applications, we believe that it is imperative to correct the basis of some of their claims: There is currently no empirical support to suggest that large effects are favored or demanded.

## Claims That Small Effects Can Be Important and Consequential Requires Empirical Evidence

Götz et al. (2022) stated further that “some small effects may also have direct real-world consequences (Funder & Ozer, 2019; Gelman & Carlin, 2014). This phenomenon is especially true for effects that accumulate over time and at scale” (p. 206). To support this claim, Götz et al. cited research on the Implicit Association Test (IAT; Greenwald et al., 2015), which claims precisely such accumulation. However, IAT researchers have been unable to provide empirical evidence for this accumulation and do not theoretically specify how such accumulation may occur (Connor & Evers, 2020). Any argument for the accumulation of a small effect must therefore be substantiated by empirical evidence rather than speculation or, at least, be supported by a falsifiable line of reasoning. This should also consider any possible mechanisms that may act against such accumulation (e.g., habituation; Anvari et al., 2021; Funder & Ozer, 2019) as well as those that facilitate it.

To further illustrate the claim that small effects can be consequential in large samples or at the population level, Götz et al. (2022) presented the correlation between aspirin and the prevention of heart attacks (*r* = .03). Ferguson (2009) pointed out the flaw in using this effect size to make the generalized argument that small effects matter through an analogy with wearing a bulletproof vest: The effect size of wearing a bulletproof vest on the probability of dying is large if we examine people who get shot but very small if we include the millions of people who never get shot. Likewise, the causal effect of aspirin on the chance of a heart attack is substantial, but there is only a small effect in the reduction of heart attacks if a large group of people, many of which would never suffer a heart attack, regularly take aspirin. The important difference between medicine and psychology is that in psychology researchers rarely include a large majority of individuals in their studies that are not expected to benefit from an intervention. For example, when we examine the effectiveness of a new treatment for depression, we usually do not conduct the study on a sample in which only a small minority of individuals are depressed. Therefore, when a small effect is observed in psychology, it may not matter at the population level, and any claims of why it would matter need to be theoretically justified and/or empirically supported.

Categorizing *r* = .03 as small regardless of empirical context, discipline, study design, and outcome variable is, in short, nonsensical. If an intervention saves hundreds of thousands of lives, then its effect on human health and society is by no reasonable definition small. If the cost of the intervention is as low as an aspirin, it is likely worthwhile to implement in practice.^2^ To judge an effect meaningful, one needs to provide evidence and a line of falsifiable reasoning.

## Summary and Discussion

Götz et al. (2022) stated that “only once small effects are accepted as the norm, rather than the exception, can a reliable and reproducible cumulative psychological science be built” (p. 205). They claimed that (a) psychology, like genetics, consists of complex phenomena explained by additive small effects; (b) psychology should not only reward large effects; and (c) small effects become meaningful at scale and over time. In this reply, we presented counterarguments outlining (a) that we cannot currently make claims about the size of effects influencing psychological phenomena in the same way as genetics, (b) that statistical significance and not effect sizes underpin publication bias and QRPs, and (c) that claims that small effects are important at scale or over time must be supported by empirical evidence and a falsifiable line of reasoning. We suggest that researchers must evaluate the meaningfulness of an effect size in respect to its theoretical and empirical context.

We argue that researchers should move away from interpreting effect sizes in an absolute manner: That is, there are no small or large effects in isolation of their contextual factors. Researchers should therefore adopt a *relative* framework to effect-size interpretation, in which the size of an effect is compared with its costs (i.e., practical or substantive significance; Kelley & Preacher, 2012; Silan, 2020), other effects in the same empirical context (e.g., this treatment effect is larger than effect sizes of other treatments), or a benchmark such as the smallest effect size of interest or maximal positive control that is established through appropriate empirics, theory, or falsifiable justification (see Anvari & Lakens, 2021; Hilgard, 2021; Rocca & Yarkoni, 2021).

Ultimately, statements about effect sizes cannot be reduced to a mechanical process, and researchers need to provide arguments that support why any effect, of any size, should be considered relevant. As psychologists start to collect larger sample sizes and restrict flexibility in their statistical analyses through the adoption of open-science practices, they will observe more accurate effect-size estimates. We are concerned that researchers confronted with very small but statistically significant effect-size estimates will cite Götz et al. (2022) as a blanket defense for why any or all small effects matter, and indeed, we are already witnessing signs of this (see Dickey et al., 2021; Greenberg et al., 2022; Jokela, 2021; Rimfeld et al., 2021; Sorlie et al., 2022). Instead, we urge researchers to justify their effect sizes and to think about the practical significance of these effects, a practice that is likely to differ between disciplines and research fields.

## Transparency

*Action Editor:* Laura A. King

*Editor:* Laura A. King

*Author Contributions*

M. A. Primbs, C. R. Pennington, D. Lakens, M. A. A. Silan, D. S. N. Lieck, P. S. Forscher, E. M. Buchanan, and S. J. Westwood conceptualized the manuscript; M. A. Primbs, C. R. Pennington, D. Lakens, M. A. A. Silan, D. S. N. Lieck, P. S. Forscher, and E. M. Buchanan wrote the original manuscript; M. A. Primbs, C. R. Pennington, S. J. Westwood, D. Lakens, M. A. A. Silan, D. S. N. Lieck, E. M. Buchanan, and P. S. Forscher reviewed and edited the manuscript; M. A. Primbs, C. R. Pennington, and D. Lakens supervised the project; and M. A. Primbs and C. R. Pennington administered the project. All of the authors approved the final manuscript for submission.

## Notes

1. We use the term “genetics” because it was used by Götz et al. (2022); we recognize that the practices outlined by Götz et al. more resemble those used in polygenic modeling of genome-wide association studies and may not be applicable to other areas of genetics.

2. But see https://www.uspreventiveservicestaskforce.org/uspstf/announcements/public-comment-draft-recommendation-statement-draft-evidence-review-and-draft-modeling-report-aspirin-use-prevent-cardiovascular. Further, as pointed out by Robert Calin-Jageman, the effect is more interpretable expressed as an odds ratio.
